# Valorization of By-Products for Functional Ingredients in Meat and Meat Replacers: A Circular Bioeconomy Approach

**DOI:** 10.3390/foods15091567

**Published:** 2026-05-01

**Authors:** Ana Leite, Lia Vasconcelos, Alfredo Teixeira, Sandra S. Q. Rodrigues

**Affiliations:** CIMO, LASusTEC, Instituto Politécnico de Bragança, Campus de Santa Apolónia, 5300-253 Bragança, Portugal; anaisabel.leite@ipb.pt (A.L.); lia.vasconcelos@ipb.pt (L.V.); teixeira@ipb.pt (A.T.)

**Keywords:** valorization, by-products, circular economy, sustainability

## Abstract

To address the pressing dual challenge of meeting global protein demand while mitigating environmental impacts, the food sector must transition to a circular bioeconomy. In this context, this review comprehensively examines the valorization of plant and animal byproducts, emphasizing how the recovery and application of their inherent bioactive and functional compounds can transform waste into high-value resources. Plant processing residues, such as fruit peels and pomace, and animal residues, such as blood and bones, are increasingly recognized as untapped sources of functional ingredients. These by-products yield bioactive compounds with health benefits. Simultaneously, the same or different compounds serve as structural building blocks, offering valuable technological properties. They improve water-holding capacity, texture, and emulsion stability in both traditional meats and plant-based analogs. While upcycling these materials reduces disposal costs and formulation expenses, challenges remain regarding compositional variability, regulatory barriers, and consumer perception of “waste-derived” ingredients. Ultimately, integrating advanced processing technologies such as enzymatic hydrolysis and fermentation is essential to building a resilient, sustainable, and circular global food system.

## 1. Introduction

The global agri-food sector is currently facing the dual challenge of meeting the nutritional demands of a rapidly growing population while mitigating the severe environmental impacts of food production. The global population is projected to reach 8.6 billion in 2030, 9.8 billion by 2050, and approximately 11.2 billion by 2100, according to a United Nations (UN) report. These projections account for mortality rates and the ongoing decline in birth rates. Rising incomes in low- and middle-income countries are expected to accelerate a shift in dietary patterns, increasing consumption of all basic resources (meat, vegetables, and cereals). This transition will require corresponding adjustments in agricultural production and may intensify the pressure placed on natural resources [1,2]. Paradoxically, thousands of tons of food are wasted daily across the supply chain, exacerbating environmental degradation. At the same time, thousands of people suffer from malnutrition or die of hunger.

In response to this global imbalance, initiatives such as the United Nations’ Zero Hunger goal and the 2030 Agenda for Sustainable Development emphasize responsible food consumption and production [3]. Within this context, the valorization of agri-food waste containing bioactive compounds has become a growing trend applicable to most industries. This approach is further supported by the Circular Economy Action Plan approved in Europe, which aims to promote the reuse of waste to obtain high-value products [4]. As a consequence of current production practices across the food industry, substantial volumes of processing residues are generated, ranging from fruit and vegetable peels and pomaces to animal co-products such as blood plasma and bone-derived fractions [5,6]. Commonly viewed as waste, these materials contribute to high disposal costs and environmental pollution. The shift towards sustainable food systems has heightened the necessity of rethinking food industry by-products, viewing them not as waste but as valuable secondary resources. In the context of the circular bioeconomy, transforming agro-industrial by-products into functional food ingredients offers a strategic approach to lessen environmental impacts while enhancing economic and nutritional value. Producing alternative proteins from waste significantly reduces the environmental footprint by requiring less land, water, and energy. This reduction is reflected in greenhouse gas emissions, which remain high in traditional animal protein production systems [7]. Analyses of food waste recovery show that reuse and conversion to produce functional ingredients support sustainable production strategies that account for food waste and material inefficiencies [8,9].

Recent studies have shown that food processing residues, including those from meat, dairy, and plant-based industries, contain substantial amounts of recoverable proteins, peptides, dietary fibers, and bioactive compounds, some of which exhibit antimicrobial activity, offering both technological and health benefits [8]. Some of these food processing residues are abundant sources of bioactive compounds of great interest to the animal and food industries, such as tocopherols, ascorbic acid, terpenes, phenolic acids, and polyphenols. They exhibit antioxidant and antimicrobial activities and can be used as alternatives to conventional preservatives in these meat matrices [9]. Also, some phytochemicals exhibit prebiotic, anti-inflammatory, antiproliferative, anti-obesity, and antidiabetic effects, among others [10].

This, coupled with improved nutritional and sensory profiles, suggests that consumers are more likely to accept and potentially prefer these enhanced products [4,11]. Another important factor in choosing these products relates to cost, the impact on animal welfare, and familiarity with the taste [12]. This opens avenues for food manufacturers to innovate and differentiate their products in the market [4,11]. However, caution and strategy are necessary when using claims such as “healthy,” “natural,” “additive-free,” “minimally processed,” “authentic,” and others on product labels, to avoid conveying a sense of trendiness or misleading consumers [9].

A circular bioeconomy approach seeks to close the loop in food systems by upcycling residual biomass into high-value components. This strategy not only reduces waste but also creates new economic streams and improves the overall resource efficiency of the food supply chain [13]. Residual biomass is increasingly recognized as an untapped reservoir that can enhance food quality, nutrition, and sustainability [6,13]. Valorizing these streams involves extracting or modifying bioactive compounds, such as fibers, proteins, and polyphenols, to serve specific technological roles in food matrices.

The rapid growth of plant-based meat analogs has intensified the search for sustainable, functional ingredient sources. Plant-derived by-products, including berry pomace, citrus peels, oilseed press cakes, fiber-rich residues, and polyphenol compounds (including flavonoids and non-flavonoids), have been investigated for their versatile techno-functional contributions and improving shelf-life stability of meat products and meat substitutes [11,14,15]. They encompass water-holding capacity (WHC), gelation, texture modulation, oxidative stability, and coloring agents [16].

Also, these plant-derived fractions provide dietary fiber and natural antioxidants that can extend shelf life and encourage consumption of reformulated products through “clean” labeling [5,9,17]. For instance, pineapple pomace and oilseed cakes (e.g., sunflower and flaxseed) have been shown to modulate the texture and chewiness of extruded meat analogs, creating structures that more closely resemble traditional muscle tissue [5,6]. Although research on valorized animal by-products in plant-based systems remains limited, the broader concept of by-product recovery for functional applications spans both conventional and alternative protein sectors.

In parallel, these ingredients (fruit pomace) are particularly valuable for addressing common quality issues in the meat industry, such as lipid oxidation, moisture loss, and a lack of dietary fiber [14,17]. However, it is important to note that although plant-based proteins have proven more efficient in terms of alternative resources, their production is susceptible to climate change and deficiencies in essential amino acids and vitamins, such as B12, highlighting the need to compensate for these deficiencies with other sources [7]. In the meat industry, significant quantities of trimmings, connective tissues, bones, blood fractions, and organ materials are produced during processing. While some of these components have traditionally been repurposed for animal feed or low-value rendering applications, recent biotechnological advancements have facilitated their conversion into high-value functional ingredients. Like vegetable by-products, animal co-products, such as porcine plasma and blood-derived proteins, offer unique gelation and binding properties and a beneficial amino acid profile, which are critical for maintaining juiciness, yield, and functionality in processed meat systems [18,19]. The application of these recycled ingredients enables a comparison between traditional meat and meat substitutes from a functional perspective. While in traditional meat, by-products often act as shelf-life extenders and nutritional enhancers, in meat substitutes they function at the level of texture, water retention capacity, and structural integrity [11,15]. Furthermore, they reinforce the viability of these valorization strategies, demonstrating potential to reduce environmental impacts and disposal costs [6,13].

Thus, a range of technologies have been developed to convert these waste products into high-value protein sources. These technologies include lignocellulosic biomass processing, single-cell proteins derived from microorganisms, insect-derived protein, and other microbial proteins cultivated on low-cost substrates. Enzymatic hydrolysis has been extensively studied as a technique to produce protein hydrolysates with enhanced solubility, emulsifying capacity, antioxidant activity, and potential bioactivity [20]. Similarly, controlled fermentation processes have demonstrated the ability to generate peptides with functional and biological significance from meat and dairy by-products, underscoring the role of microbial biotechnology in waste valorization [21]. These technological advancements align with circular economic principles by preserving material value throughout the food chain. Despite the growing volume of scientific research in specific areas, such as the use of animal by-products and the development of plant-based ingredients, this knowledge remains fragmented. Notably, there is a clear lack of comprehensive reviews that integrate both meat and meat replacer systems within a cohesive circular bioeconomy framework.

This review aims to explore ways of recovering plant by-products and animal co-products as functional ingredients within the framework of a circular bioeconomy. It provides examples of potential applications based on their technological properties and examines their comparative functionality in both traditional meat products and meat substitutes. By highlighting the sustainability and economic benefits of these approaches, this review underscores the critical role of waste valorization in building a more resilient and circular global food system. For this review, online searches of databases such as Scopus, ScienceDirect, MDPI, PubMed, and Web of Science were performed. Inclusion criteria included strings like: “application in meat products,” “application in meat analogs,” and exclusion criteria: “if the inclusion criteria were not met,” if the papers were published more than 20 years ago, unless they were highly relevant. The search used the following keywords and strings: “meat replacers applications,” “meat functional ingredients,” “functional components,” “technological functional compounds,” “functional ingredients,” “bioactive ingredients,” “plant-origin by-products,” “animal-origin by-products,” “circular economy”.

## 2. Circular Economy and Valorization of By-Products

**Figure 1:**
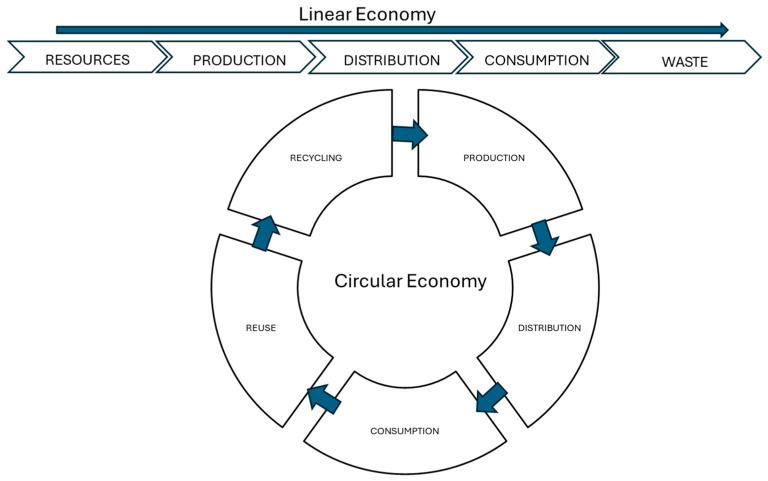
The linear and circular economy.

The urgent need to address environmental and climate challenges continues to intensify. Traditional economic models based on production and disposal are increasingly recognized as unsustainable, highlighting the need for systemic change. Growing pressure to adopt more sustainable approaches is driving the transition from a linear to a circular economy, prioritizing resource efficiency, waste reduction, and long-term value retention. According to the World Health Organization [22], the circular economy is grounded in a structural and systemic shift away from a linear economic paradigm. Whereas the linear approach is characterized by the extraction of natural resources, production, use, and eventual disposal, the circular economy aims to maintain resources in circulation for as long as possible through reuse, recycling, and recovery strategies. While the circular economy presents clear environmental advantages over the linear model, its implementation is not without trade-offs. Circular systems often require higher initial investments, more complex logistics, and advanced technologies for material recovery and processing, which may limit their feasibility across sectors. In addition, the environmental benefits of circular processes are not always guaranteed, as some recovery and transformation technologies can be energy-intensive, potentially offsetting sustainability gains [23]. The circular and linear economy models are illustrated in Figure 1.

The concept of the circular economy has also become increasingly relevant in the context of waste management and the utilization of industrial by-products. Unlike traditional waste management approaches, circular strategies intend to convert residues and by-products into valuable resources that can be reintegrated into production systems. This approach reduces environmental impacts and promotes the efficient use of materials and energy across supply chains. Agro-industrial by-products have attracted increasing attention due to their high content of bioactive compounds, fibers, and other valuable constituents that can be recovered and used in the food, pharmaceutical, and cosmetics industries. Converting these materials into value-added products aligns with the principles of the circular bioeconomy and reduces the environmental impact of waste disposal [24].

However, the large-scale implementation of by-product valorization strategies remains challenging. One of the main limitations is the variability in the composition of agro-industrial residues, which depends on factors such as raw material origin, seasonal variations, and processing conditions, complicating process standardization and industrial scalability. Furthermore, not all by-products are economically viable to valorize, as the costs of collection, transportation, processing, and quality control may exceed the market value of the recovered compounds [25]. According to the Food and Agriculture Organization, approximately one-third of all food produced globally for human consumption is lost or wasted each year, amounting to nearly 1.3 billion tons. This food is lost or wasted throughout the supply chain, including during production, processing, distribution, retail, and final consumption. In addition to the economic losses associated with wasted resources, food waste contributes significantly to environmental degradation, including greenhouse gas emissions, land degradation, and inefficient water use [26].

### Animal and Plant By-Products

The food sector is currently facing a range of structural and conjunctural challenges that significantly affect its economic, environmental, and social sustainability. Among the main challenges are rising production costs, driven by higher energy and raw material prices, as well as the impacts of geopolitical conflicts that disrupt global supply chains and logistics systems. In addition, labor shortages in several regions, combined with changing consumer habits—particularly the growing demand for healthier, more sustainable foods with transparent origins—require continuous adaptation from companies operating in the food sector. At the same time, sustainability and climate change have become central issues in the agri-food sector. Food production systems are facing an increasing number of challenges relating to resource efficiency and waste generation. In this context, the valorization of agro-industrial by-products has emerged as a key strategy to enhance sustainability, mitigate environmental impact, and foster the development of more efficient food systems [27,28].

Reducing food waste has been widely recognized as a global priority. It is estimated that a significant proportion of food produced worldwide is lost or wasted across different stages of the food supply chain, from agricultural production to final consumption. This phenomenon has a considerable economic impact due to the waste of water, energy, land, and other resources used in food production. Throughout the various stages of the food production chain, numerous by-products and residues are generated. Traditionally, many of these materials have been undervalued or directed toward low-value applications, such as animal feed or disposal. However, recent studies demonstrate that several of these by-products have high valorization potential and can be used as alternative raw materials rich in bioactive compounds, dietary fibers, proteins, and other constituents such as lipids, minerals, vitamins, and enzymes with relevant technological and functional properties. The valorization of food by-products is closely aligned with the circular economy, which aims to maximize resource efficiency and minimize waste generation across the entire production system. In this sense, transforming by-products into value-added ingredients or materials of the food sector, while simultaneously contributing to waste reduction and innovation in the development of new food products [29], represents a vital strategy.

Animal and plant by-products are generated in large quantities throughout the food production and processing chain. These materials originate from agricultural activities, food processing operations, and industrial processes, and generally include husks, seeds, pomace, skins, bones, and other residual fractions. Although traditionally considered waste or used in low-value applications such as animal feed or disposal, these by-products have been attracting increasing scientific and industrial interest due to their rich composition of bioactive compounds, dietary fiber, proteins, lipids, and other functional components. The recovery and valorization of these materials represent important strategies for improving resource efficiency, reducing environmental impact, and promoting more sustainable and circular food systems [9].

Despite growing scientific and industrial interest in valorizing plant and animal by-products, several factors still limit their large-scale implementation. One of the main challenges is the variability in the chemical composition of these materials, which can depend on factors such as species, processing conditions, and seasonal variations. Additionally, technological limitations in extraction, stabilization, and processing can hinder the efficient recovery of valuable compounds. Furthermore, regulatory constraints on incorporating recovered ingredients into food products represent an important limitation, as strict safety and quality standards must be met. Furthermore, consumer perception of foods derived from waste streams may influence market acceptance, underscoring the importance of transparency and communication about the sustainability benefits of these practices.

These challenges require the development of standardized, efficient processing technologies and supportive regulatory frameworks that facilitate the safe use of recovered compounds in food systems. Increasing research efforts and greater consumer awareness are also essential to promoting the transition toward more sustainable, circular food production systems. In this context, valorizing plant and animal by-products is a promising strategy to improve resource efficiency, reduce environmental impacts, and support the development of innovative, sustainable food products.

## 3. Functional Compounds Derived from By-Products

A functional compound—often referred to as a bioactive compound or functional ingredient—is defined as a specific chemical substance found in, or extracted from, a biological source that provides a targeted benefit when added to a food matrix [30,31]. Within the context of meat and meat analogs, these compounds generally fall into two distinct categories based on their intended target: those that provide physiological health benefits to consumers and those that serve technological functions within the food structure.

### 3.1. Bioactive Functional Compounds (Health-Targeted)

Bioactive functional compounds are molecules that confer physiological health benefits beyond basic nutritional requirements. They are primarily utilized in the formulation of functional foods intended to promote optimal health or mitigate the risk of chronic diseases. A diverse array of these compounds, derived from both plant and animal sources [32,33], can be integrated into meat products and their analogs to enhance their nutritional profiles.

#### 3.1.1. Plant-Derived Bioactive Compounds

Plants, fruits, vegetables, and their processing by-products are rich reservoirs of functional compounds that offer significant health benefits, primarily by protecting against cellular oxidative damage. Polyphenols, encompassing a wide array of flavonoids [34] and phenolic acids (e.g., gallic, caffeic, and salicylic acids) [35], act as powerful antioxidants and anti-inflammatory agents. These compounds provide extensive systemic benefits, including cardioprotective and neuroprotective effects, as well as improved endothelial function. Similarly, carotenoids (such as β-carotene, lycopene, and lutein) are highly valued for their strong antioxidant, anti-inflammatory, and anti-carcinogenic properties [36]. Glucosinolates—precursors to active compounds like sulforaphane and isothiocyanates—further contribute to disease prevention by supporting natural detoxification pathways [37]. Finally, essential vitamins found in these plant sources, such as C, A, K, and folate, play a foundational role in general disease prevention, management, and overall health.

In addition to the previously mentioned compounds, plant sterols (which include both sterols and stanols) play a vital role in health management. They are specifically noted for their ability to aid in cholesterol and cancer control, while contributing to a broader One Health approach [38]. Naturally present in ruminant meat, conjugated linoleic acid (CLA) of plant origin has been used to enrich meat products [39], which are valued for their ability to regulate immune responses and support lipid metabolism [40]. Furthermore, dietary fiber, encompassing both soluble and insoluble forms, offers significant functional and technological benefits. These include prebiotic dietary fibers (such as various oligosaccharides, polysaccharides, and resistant starches), which stimulate beneficial gut microbiota [41], as well as dietary fibers that reduce cholesterol levels, blood pressure, and inflammation, and improve vascular and immune function [42]. Beyond basic nutritional fortification, dietary fibers are highly effective tools for replacing fat and reducing calories, making them incredibly useful for creating healthier, lower-fat food formulations without sacrificing texture.

Table 1 shows different plant-derived functional compounds and their bioactive effects.

#### 3.1.2. Animal-Derived Bioactive Compounds

Animal-derived functional compounds (Table 2) play a dual role in modern food science: they enhance the physiological benefits of traditional meat products and serve as critical fortifying agents to address nutritional gaps in plant-based meat analogs, thereby transforming both into targeted functional foods. Based on their biological applications, they can be grouped into different categories.

Derived from the breakdown of animal proteins, bioactive peptides obtained from the fermentation of various sources, such as shrimp waste, bovine, porcine, and poultry byproducts, act as powerful dual-purpose ingredients. Technologically, their natural antioxidant and antimicrobial properties help delay lipid oxidation and inhibit the growth of foodborne pathogens, thereby vastly extending the shelf life of the meat matrix. Physiologically, they provide targeted health benefits, most notably serving as antihypertensive agents and immune system enhancers [54,55]. Expanding on peptide technology, fermented dairy bioactives (probiotic metabolites, antimicrobial peptides, and immunomodulatory peptides) derived from dairy fermentation [56] provide significant gut health support, immune enhancement, and systemic anti-inflammatory benefits.

Lipid fractions, such as Omega-3 polyunsaturated fatty acids (PUFAs)—specifically DHA (docosahexaenoic acid), and EPA (eicosapentaenoic acid)—obtained from fish oil [57], are increasingly incorporated into meat products to improve their lipid profiles. Omega-3s are fortified for their cardioprotective, anti-inflammatory, and neuroprotective properties [58]. Due to their susceptibility to oxidation, these highly unsaturated fats often require microencapsulation prior to incorporation [51]. Furthermore, naturally occurring endogenous antioxidants (Coenzyme Q10, Glutathione, and Lipoic Acid), provide potent systemic anti-inflammatory properties while serving as critical technological aids by scavenging free radicals and preserving myoglobin color [59,60,61].

Beyond their vital technological role as emulsifier (such as lecithin, which stabilizes the fat–water matrix in vegan burgers), choline, found in various animal products and byproducts such as liver, kidneys, heart, dairy fractions, and egg yolk [62], acts as a potent biological regulator. It actively supports neurotransmitter synthesis for optimal cognitive function and plays a foundational role in liver health [63].

Crucially, modern plant-based meat analogs often require fortification with “carninutrients”—bioactive compounds that are naturally abundant in animal tissues but absent in the plant kingdom—to achieve true nutritional mimicry [64]. These include creatine, which acts as a vital cellular energy buffer; L-carnitine, which facilitates fatty-acid transport for cellular energy production; and taurine, which provides essential cardioprotective and neurological support.

**Table 2:** Functional compounds with animal origin and their effects.

Compound Category	Examples	Functional Effects	References
Bioactive Peptides	Antihypertensive peptides, antioxidant peptides, antimicrobial peptides	Antioxidant, antihypertensive, antimicrobial, immune-enhancing	[65,66,67,68]
Omega 3 Long-Chain Fatty Acids	DHA, EPA	Cardioprotective, anti-inflammatory, neuroprotective	[69]
Endogenous Antioxidants	Coenzyme Q10, glutathione, lipoic acid	Antioxidant, anti-inflammatory, supports energy production	[59,60,61]
Fermented Dairy Bioactives	Probiotic metabolites, antimicrobial peptides, immunomodulatory peptides	Gut-health support, immune enhancement, anti-inflammatory	[56,70,71,72,73,74,75]
Choline & Related Compounds	Choline, betaine	Supports neurotransmitter synthesis, liver health, cognitive function	[63,76]
Creatine (in meat analogs)	Creatine, phosphocreatine	Enhances energy metabolism; supports muscle performance and brain function	[64,70]
Carnitine (in meat analogs)	L carnitine	Facilitates fatty-acid transport for energy production; contributes to overall metabolic and muscle function	[71]
Taurine (in meat analogs)	Taurine	Cardioprotective, antioxidant, and neurological support	[70]

### 3.2. Technological Functional Compounds (Matrix-Targeted)

Bioactive compounds primarily target human health. In contrast, technological functional compounds are utilized specifically to solve structural and manufacturing challenges, such as binding moisture, stabilizing emulsions, extending shelf life, and engineering precise textural attributes. Based on their biological origin, these technological tools function in distinct ways across the meat industry.

#### 3.2.1. Plant-Origin Technological Compounds

Because plant-based meat analogs inherently lack the myofibrillar proteins that confer gelling in animal muscle, food scientists must construct the meat matrix using plant-derived ingredients [77]. Functional plant proteins (e.g., highly extruded wheat gluten or soy isolate) provide the fundamental fibrous texture, while plant-based emulsifiers (e.g., soy lecithin) are required to bind the added plant oils and water, preventing fat separation during thermal processing [78,79].

Hydrocolloids, such as carrageenan, xanthan gum, and konjac, are widely used for their exceptional water-binding and gel-forming properties [80]. In traditional meat products, dietary fibers act as moisture-retaining agents, enabling significant reductions in animal fat while maintaining a juicy mouthfeel [53]. Additionally, natural plant extracts (such as rosemary and oregano) serve as potent antioxidants that inhibit lipid oxidation [81], while nitrate-rich vegetable powders (like celery juice powder) act as clean-label curing agents to stabilize the classic pink pigment of processed meats [82].

Table 3 presents plant-origin technological compounds and their effects on meat products and their analogs.

#### 3.2.2. Animal-Origin Technological Compounds

In the traditional meat sector, animal-derived compounds are highly prized for their unparalleled binding strength and thermo-reversible properties [85,86]. Dairy proteins, specifically sodium caseinate, act as highly effective emulsifiers, rapidly coating fat droplets to create stable oil-in-water emulsions that withstand high cooking temperatures [87,88]. Ingredients like gelatin provide strong cold-set gels ideal for slicing deli meats, while egg whites form firm heat-set gels upon cooking. Finally, cross-linking enzymes, such as microbial transglutaminase, catalyze covalent bonds between protein molecules, allowing processors to restructure meat trimmings and improve product texture [89,90].

Table 4 shows animal-origin technological compounds and their effects on meat products and their analogs.

Although these functional ingredients offer significant advantages, their high extraction costs often limit universal commercial application. Consequently, they are typically reserved for premium health markets or necessary fortification in meat analogs. However, embracing the circular bioeconomy presents a sustainable economic solution [98]. By upcycling underutilized animal by-products (such as blood plasma, viscera, and dairy processing streams) and plant side streams (such as oilseed press cakes and fruit pomace), the food industry can transform low-value raw materials into premium functional ingredients [99,100]. This approach not only minimizes global agricultural waste but drastically lowers formulation costs, creating an economically viable and nutritionally robust supply chain for both traditional and alternative meat sectors. Ultimately, the most valuable functional compounds within this framework are those exhibiting dual functionality—such as apple pomace dietary fiber, which acts technologically to bind water while physiologically lowering glycemic responses [17,101,102,103].

## 4. Application

This section describes the practical application of functional compounds derived from agro-industrial by-products in meat products and plant-based meat analogs. The discussion focuses on their technological functionality, contribution to product quality, and relevance within a circular bioeconomy framework. Because of this broad scope and the highly variable methodologies and matrices used across the cited literature, direct quantitative comparisons have been omitted to avoid misrepresentation, focusing instead on overarching qualitative trends.

In the meat industry, by-products such as blood, skin, bones, and connective tissues are increasingly utilized as sources of functional ingredients. These materials provide proteins (e.g., collagen, gelatin, and blood proteins) with well-established technological properties, particularly in emulsified and restructured products such as sausages and pâtés. Their incorporation improves water-holding capacity, emulsion stability, texture, and product yield.

**Figure 2:**
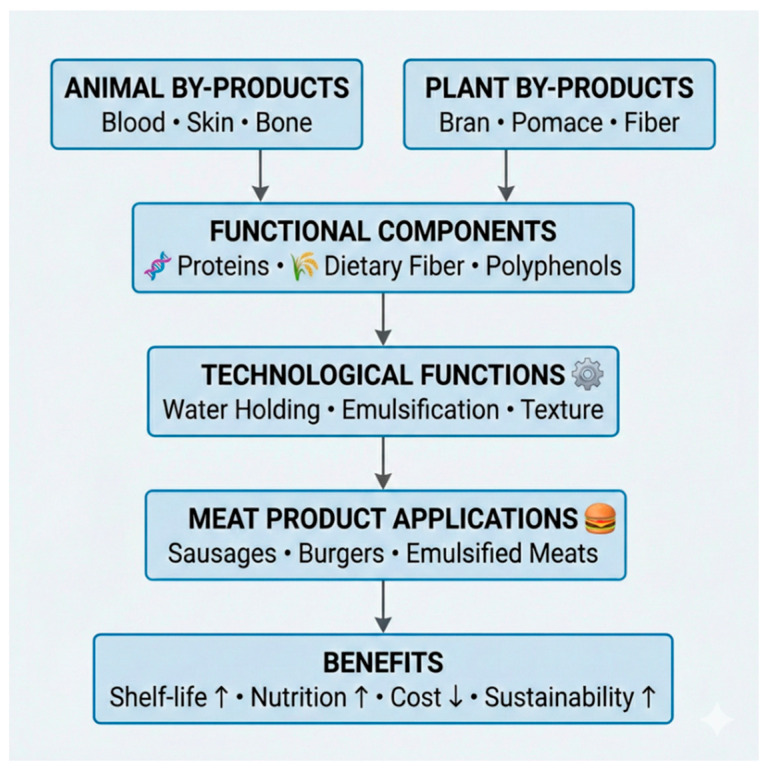
Valorization of by-products as functional ingredients in meat products.

The main sources, functional components, and applications of by-products in meat systems are summarized in Figure 2.

Plant-derived by-products, including fruit pomace, vegetable peels, and cereal bran, are also widely incorporated into meat formulations. Due to their high content of dietary fiber and polyphenols, these ingredients contribute to oxidative stability, reduce cooking losses, and enable partial replacement of fat and synthetic additives. Consequently, they support the development of products with improved nutritional profiles and cleaner labels. Historically considered low-value materials or waste, these by-products are increasingly recognized as valuable sources of functional ingredients with technological, nutritional, and economic benefits. Within the framework of the circular bioeconomy, their valorization enables reintegration into the food chain, reducing waste and improving resource efficiency [104,105] while supporting the transition toward more sustainable and circular food systems [8,106].

In plant-based meat analogs, by-products serve as sustainable, cost-effective sources of structuring agents and bioactive compounds. Fiber-rich residues (e.g., brewer’s spent grain, soy pulp, and vegetable fibers) contribute to the formation of fibrous structures that mimic the texture of muscle tissue, while plant proteins provide the structural matrix required for product integrity. Similarly, collagen extracted from skin and connective tissues contributes to improved texture, binding, and structural integrity in processed meats [98,106,107].

In addition, the antioxidant activity of plant-derived phenolics plays a crucial role in delaying lipid oxidation, thereby extending the shelf life of meat products and maintaining their sensory quality [9,108,109]. This mechanism is particularly valuable in the reformulation of low-fat meat products, where fibers can partially replace animal fat while maintaining desirable texture and juiciness. In meat analogs, fiber-rich residues such as sweet potato fiber, brewer’s spent grain, or soy pulp contribute to the development of fibrous structures that mimic the anisotropic texture of muscle tissue.

Animal-derived compounds also provide important technological advantages in meat processing. Collagen, gelatin, and bioactive peptides extracted from skin, bones, and connective tissues are extensively used as structuring agents due to their thermo-reversible gelling properties. These proteins form stable three-dimensional networks capable of improving binding, elasticity, and sliceability in emulsified meat products such as sausages, pâtés, and deli meats. Similarly, blood-derived proteins exhibit strong emulsifying and foaming properties that stabilize fat–water emulsions in processed meat systems.

These compounds are widely used to improve texture, nutritional value, and functional performance in meat products [9,106]. In addition, emerging sources such as algae residues, fermentation biomass, and insect-derived materials are gaining relevance due to their high protein content and sustainability potential. Bioactive peptides obtained through enzymatic hydrolysis of meat or dairy by-products provide additional functional benefits. Beyond their technological roles as antioxidant and antimicrobial agents that extend product shelf life, these peptides contribute to the development of functional foods by offering physiological benefits such as antihypertensive and immune-modulating effects. Their incorporation into processed meats or hybrid products, therefore, supports both product stability and nutritional enhancement. Technological functional compounds are equally essential for structuring plant-based meat analogs. Plant proteins such as soy isolate, pea protein, and wheat gluten form the fundamental structural network responsible for the fibrous texture of meat substitutes. Hydrocolloids derived from plant sources (e.g., carrageenan, xanthan gum, and konjac) further reinforce this structure by improving gel formation and water retention. These ingredients compensate for the absence of myofibrillar proteins in plant-based matrices and help reproduce the juiciness, bite, and cohesiveness characteristic of conventional meat products. The incorporation of by-products into meat products offers multiple functional benefits. From a technological perspective, these ingredients enhance water-holding capacity, emulsion stability, and fat-binding properties, thereby improving texture and product yield. Dietary fibers and protein extracts derived from by-products can act as structuring agents, reducing formulation costs while maintaining product quality. Moreover, the presence of polyphenols and other antioxidant compounds contributes to oxidative stability by delaying lipid and protein oxidation, which are primary causes of quality deterioration in meat products [9]. In addition to antioxidant effects, certain by-products exhibit antimicrobial properties due to the presence of phenolic compounds and bioactive peptides, thereby extending shelf life and improving food safety.

Nutritionally, the use of by-products enriches meat products with dietary fiber, micronutrients, and bioactive compounds, aligning with current consumer demand for healthier foods. Furthermore, these ingredients support clean-label formulations by replacing synthetic additives such as preservatives and stabilizers with natural alternatives. This is particularly important in the context of increasing consumer awareness and preference for minimally processed foods with recognizable ingredients.

In parallel, by-products are increasingly utilized in the development of meat replacers, particularly plant-based analogs. These products rely on plant proteins and structuring agents to mimic meat’s sensory characteristics. By-products such as oilseed meals and legume residues serve as cost-effective protein sources, while dietary fibers contribute to the development of fibrous textures and improved mouthfeel [16]. Additionally, by-products can enhance sensory properties, such as flavor and color, though careful formulation is required to avoid undesirable attributes. The presence of bioactive compounds in plant-derived by-products further contributes to the development of functional meat analogs with potential health benefits, such as antioxidant and anti-inflammatory effects.

Advanced processing technologies play a crucial role in enabling the use of by-products in both meat products and meat replacers. Techniques such as extrusion are widely used to create fibrous structures in plant-based meat analogs, while enzymatic hydrolysis and fermentation improve digestibility, functionality, and flavor. These processes are essential for overcoming some of the limitations associated with raw by-products, including poor sensory properties and limited nutrient bioavailability.

From a circular bioeconomy perspective, the valorization of by-products represents a key strategy for achieving sustainable food systems. By converting waste streams into functional ingredients, the food industry can reduce environmental impacts, lower greenhouse gas emissions, and decrease reliance on primary raw materials [104,106]. Industrial symbiosis further enhances this approach by facilitating the exchange of by-products between sectors, such as the use of brewery residues in food formulations or the transformation of dairy whey into protein ingredients. The biorefinery concept extends this idea by integrating multiple processes to extract high-value compounds from biomass while minimizing waste generation [109].

Despite these advantages, several challenges must be addressed to fully exploit the potential of by-products in food applications. Safety concerns, including the presence of contaminants such as mycotoxins and heavy metals, require careful monitoring and control. Additionally, variability in by-product composition, driven by seasonal and processing differences, poses challenges for standardization and consistent product quality.

To better illustrate the practical implementation of these compounds, Table 5 summarizes representative applications of agro-industrial by-products and their derived functional compounds in meat products and plant-based meat analogs. The table highlights the origin of the by-product, the main functional compounds obtained, and their technological or nutritional role in the final product. These examples demonstrate how bioactive compounds recovered from plant and animal residues can be strategically incorporated into food formulations to improve product quality, enhance shelf life, and contribute to the development of sustainable food systems aligned with circular bioeconomy principles.

Processing technologies play a key role in enabling the efficient utilization of by-products. Extrusion is widely used to develop structured plant-based products, while enzymatic hydrolysis and fermentation improve the functionality, digestibility, and bioavailability of compounds derived from both plant and animal sources. These approaches contribute to overcoming limitations associated with raw by-products, including variability in composition and suboptimal sensory characteristics.

The incorporation of by-products into meat products and meat analogs provides several functional advantages:

- Improvement of technological properties, including water-holding capacity, emulsion stability, texture, and yield;
- Enhancement of nutritional value through the addition of dietary fiber, bioactive compounds, and micronutrients;
- Extension of shelf life due to antioxidant and antimicrobial activity.
- Contribution to sustainability by reducing waste and improving resource efficiency.

Despite these advantages, several limitations remain. Variability in raw material composition may affect process standardization and product consistency. Safety concerns, including the potential presence of contaminants, require strict monitoring and quality control. In addition, sensory limitations (e.g., off-flavors, color changes, and texture alterations) may restrict the level of incorporation. Regulatory constraints and consumer perception of by-products as “waste-derived” ingredients also represent important barriers to industrial implementation.

A synthesis of the applications presented in Table 5 indicates that agro-industrial by-products provide multifunctional benefits across both meat products and meat analogs, particularly in terms of water-holding capacity, emulsion stability, and textural enhancement. However, further research is required to improve standardization, sensory quality, and scalability to support their broader industrial adoption.

## 5. Future Perspectives

The valorization of agro-industrial by-products is a key strategy for the development of sustainable food systems aligned with circular bioeconomy principles. However, further research and technological advancements are required to support large-scale implementation.

Future studies should focus on optimizing extraction and processing technologies to improve efficiency, scalability, and economic feasibility. Approaches such as biorefinery systems, precision fermentation, and enzyme-assisted processing are expected to enhance the recovery and functionality of valuable compounds. In addition, the application of artificial intelligence and data-driven tools may support formulation optimization and process control.

Ensuring the consistent quality of by-product-derived ingredients remains a major challenge. Standardized processing protocols and quality control systems are required to minimize variability. Furthermore, comprehensive safety assessments are necessary to ensure compliance with regulatory requirements and to prevent contamination by undesirable compounds.

Although technological functionality has been widely demonstrated, future research should increasingly address sensory quality and consumer acceptance. Improvements in flavor, texture, and appearance are essential, particularly at higher inclusion levels. The development of hybrid products combining animal and plant ingredients may represent a promising strategy to enhance acceptability.

Consumer perception plays a critical role in the adoption of by-product-derived ingredients. Clear communication, transparent labeling, and increased awareness of sustainability benefits are necessary to improve acceptance. Shifting the perception from “waste-derived” to “value-added” ingredients is essential for market success.

The establishment of harmonized regulatory frameworks will facilitate the safe use of by-products in food applications. In parallel, environmental and economic assessments, including life cycle assessment and techno-economic analysis, are required to quantify the sustainability benefits of valorization strategies.

The integration of by-product-derived ingredients into meat products and meat analogs represents a promising pathway toward more sustainable and efficient food systems. Continued progress will depend on advances in processing technologies, regulatory support, and consumer engagement. These efforts will enable the transformation of underutilized resources into high-value functional ingredients, supporting innovation in the food industry.

## Figures and Tables

**Table 1:** Functional compounds found in plants, fruits and vegetables, and their effects.

Category	Examples	Functional Effects	Reference
Polyphenols	Flavonoids (flavones, flavonols, isoflavones, flavanones, and anthocyanins) Phenolic acids (hydroxybenzoic (salicylic acid, protocatechuic acid, vanillic acid, benzoic acid, gallic acid, and ellagic acids) and hydroxycinnamic acids (p-coumaric, caffeic, ferulic, and sinapic acids)	Antioxidant, anti-inflammatory, antidiabetic, cardioprotective, neuroprotective; improve endothelial function; chronic disease risk reduction	[35,42,43,44]
Carotenoids	β-carotene, lycopene, lutein, α-carotene	Antioxidant, anti-cancer, anti-inflammation, and anti-allergenic properties	[36]
Glucosinolates	Sulforaphane precursors, isothiocyanate precursors	Anti-cancer, antioxidant, detoxification supportive	[37,45]
Vitamins	C, A, K, folate	Disease prevention, management, and control	[46]
Minerals	K, Mg, Ca	Maintaining normal health, preventing disease, optimal functioning of the immune system	[47,48]
Plant sterols	Sterols, stanols	Cholesterol control, cancer control, contribution to one health approach	[38,49,50]
CLA	c9,t11 CLA, t10,c12 CLA	Supports lipid metabolism, immune regulation, fat oxidation	[40,51]
Dietary fiber	Soluble & insoluble	Fat replacement, calorie reduction, nutritional fortification	[52,53]

**Table 3:** Plant-origin technological compounds and their effects.

Compound Category	Examples	Technological Functional Effects	Reference
Hydrocolloids & Gums	Carrageenan, xanthan gum, konjac root, alginate, agar	Gelation & Binding: Binds massive amounts of water to create firm, sliceable gels. In vegan meats, konjac and carrageenan are often used to mimic the firm-but-melting texture of animal fat pockets.	[80,83]
Dietary Fibers	Citrus fiber, oat fiber, bamboo fiber, cellulose	Moisture Retention & Fat Replacement: Acts like a microscopic sponge. It dramatically reduces “drip loss” during cooking, keeping the meat juicy, and provides bulk to replace animal fat in low-fat sausages.	[53,84]
Functional Plant Proteins	Soy protein isolate, wheat gluten (seitan), pea protein	Texturization & Emulsification: Creates the core structural network. Wheat gluten provides a highly elastic, fibrous chew, while soy and pea proteins act as structural emulsifiers to hold the batter together.	[78,79]
Natural Preservatives & Color Fixers	Rosemary extract, celery juice powder, cherry powder	Shelf Life & Curing: Rosemary extract prevents lipid oxidation (rancidity). Celery powder provides natural nitrites, which cure the meat, prevent botulism, and lock in the classic pink color of hot dogs and bacon.	[81,82]
Plant Emulsifiers	Soy lecithin, sunflower lecithin	Emulsion Stability: Prevents fat and water from separating. Ensures that the added plant oils don’t simply melt and leak out of a vegan burger when it hits a hot grill.	[79]

**Table 4:** Animal-origin technological compounds and their effects.

Compound Category	Examples	Technological Functional Effects	References
Connective Tissues & Blood Proteins	Gelatin, collagen powder, blood plasma	Cold-Set Gelation & Yield: Gelatin and collagen melt when heated but form strong, sliceable gels when chilled (crucial for deli meats and pâtés). Blood plasma is an incredibly strong binder used to glue meat particles together.	[91,92]
Dairy Proteins	Sodium caseinate, whey protein concentrate	Superior Emulsification: Caseinates are among the most powerful emulsifiers in food science. They coat fat droplets in finely milled sausages to completely prevent fat rendering (greasing out) during cooking.	[87,88]
Cross-linking Enzymes	Transglutaminase (often called “meat glue”)	Structural Restructuring: An enzyme that catalyzes permanent covalent bonds between protein molecules. It is used to bind scrap meat pieces together into uniform steaks or to give sausages a perfectly snappy “bite.”	[93,94]
Egg Derivatives	Egg white powder (ovalbumin), egg yolk lecithin	Heat-Set Gelation: Egg whites form irreversible, highly elastic gels when heated, providing a very firm chew to processed meats. Egg yolks are used for their natural emulsifying power.	[95,96]
Fermented Dairy Bioactives	Exopolysaccharides (EPS) from Lactic Acid Bacteria	Biothickening: Long-chain sugars secreted by dairy bacteria that act as natural thickeners and water-binders, improving the mouthfeel and stability of low-fat fermented meats (like salami).	[74,97]

**Table 5:** Applications of by-products as functional ingredients in meat products and meat replacers.

By Product	Ingredient Type	Application	Functional Role	Key Findings	References
Blood plasma	Animal protein	Sausages	Emulsifier, binder	Improved emulsion stability at 10% replacement	[19]
Animal skin, bones	Collagen/gelatin	Sausages, emulsified meats	Gelation, water-holding, emulsification	Improved textures, stability, and elasticity in meat systems	[110]
Blood (porcine/bovine)	Blood proteins/plasma	Meat emulsions, sausages	Emulsification, binding	Enhanced emulsion stability and protein functionality	[111]
Meat by-products (liver, skin, bones)	Protein hydrolysates/peptides	Functional meat formulations	Antioxidant, antimicrobial	Bioactive peptides improve nutritional and functional properties	[112]
Meat processing streams	Recovered proteins	Sausages	Protein replacement	Maintained quality at ≤10% inclusion	[19]
Collagen (skin/bones)	Gelatin/collagen	Emulsified meats	Gelation, WHC	Improved texture and elasticity	[113]
Wheat bran	Dietary fiber	Functional sausages	WHC, fat replacement	Improved fiber content and shelf-life	[114]
Date fiber	Plant fiber	Meat analogs	Fibrous structure	Improved anisotropy and texture	[114]
Okara	Soy by-product fiber	Pork jerky	Meat extender	Up to 10% improved texture and flavor	[115]
Okara	Protein + fiber	Burgers/sausages	Fat reduction, WHC	Increased fiber; reduced fat	[116]
Brewer’s spent grain	Insoluble fiber	Meat analogs	Texture structuring	Enhanced “meaty” texture	[117]
Vegetable residues	Fiber	Meat extenders	WHC, yield	Increased yield and reduced fat	[116]
Cereal ingredients	Plant-based ingredients	Hybrid meat sausages	Reduce meat content	Acceptable sensory properties	[118]
Mixed agro-industrial by-products	Bioactives	Functional meat	Antioxidant	Improved nutritional quality	[113]
Fruit pomace	Polyphenols + fiber	Meatballs	Antioxidant	Increased fiber and oxidative stability	[119]
Liver/offal	Organ meat	Pâté	Nutritional enrichment	Increased protein and micronutrients	[119]
Edible filamentous fungi	Protein bioingredients	Meat analogs	Functional protein source	Alternative protein source	[120]
Animal by-products (general)	Bioactive peptides	Functional foods	Antioxidant, antimicrobial	Added health functionality	[121]
Olive cake (in animal diet—indirect)	Functional feed	Dry-cured meat	Lipid profile modulation	Improved fatty acid profile and oxidative stability	[122]
Sweet potato residues	Fiber	Meat analogs	Structure	Similar texture to meat	[123]
Soy pulp residues	Fiber/protein	Hybrid sausages	Texture	Acceptable sensory profile up to 20%	[123]
Bamboo shoot residue	Fiber	Hybrid meat	Texture improvement	Improved emulsion stability	[123]
Olive cake (in animal diet—indirect)	Functional feed	Dry-cured pork meat products	Lipid quality	Lower PUFA n-6/n-3 ratio	[124]
Olive cake (in animal diet—indirect)	Functional feed	Pork meat quality	Chemical Compositions Lipid profile	Optimizing feeding strategies, better meat quality and sustainability.	[125]

## Data Availability

No new data were created or analyzed in this study.
